# Predicting Mental Health Status in Remote and Rural Farming Communities: Computational Analysis of Text-Based Counseling

**DOI:** 10.2196/33036

**Published:** 2022-06-21

**Authors:** Mark Antoniou, Dominique Estival, Christa Lam-Cassettari, Weicong Li, Anne Dwyer, Abìlio de Almeida Neto

**Affiliations:** 1 The MARCS Institute for Brain, Behaviour and Development Western Sydney University Penrith Australia; 2 Centre for Work Health and Safety New South Wales Government Gosford Australia

**Keywords:** e-mental health, text-based, counseling, Linguistic Inquiry and Word Count, LIWC, depression, anxiety, stress

## Abstract

**Background:**

Australians living in rural and remote areas are at elevated risk of mental health problems and must overcome barriers to help seeking, such as poor access, stigma, and entrenched stoicism. e-Mental health services circumvent such barriers using technology, and text-based services are particularly well suited to clients concerned with privacy and self-presentation. They allow the client to reflect on the therapy session after it has ended as the chat log is stored on their device. The text also offers researchers an opportunity to analyze language use patterns and explore how these relate to mental health status.

**Objective:**

In this project, we investigated whether computational linguistic techniques can be applied to text-based communications with the goal of identifying a client’s mental health status.

**Methods:**

Client-therapist text messages were analyzed using the Linguistic Inquiry and Word Count tool. We examined whether the resulting word counts related to the participants’ presenting problems or their self-ratings of mental health at the completion of counseling.

**Results:**

The results confirmed that word use patterns could be used to differentiate whether a client had one of the top 3 presenting problems (depression, anxiety, or stress) and, prospectively, to predict their self-rated mental health after counseling had been completed.

**Conclusions:**

These findings suggest that language use patterns are useful for both researchers and clinicians trying to identify individuals at risk of mental health problems, with potential applications in screening and targeted intervention.

## Introduction

### Rural Australians Are at Increased Risk of Mental Health Problems

Australians who live in rural and remote communities are at increased risk of adverse health outcomes because they face a combination of chronic, but unpredictable, stressors. Rural communities are those geographic areas located outside towns and cities, and remote areas are places that are isolated or considerably secluded from civilization. Overall, there are fewer employment opportunities than in urban centers, and reliance on primary industries leaves rural areas prone to financial instability owing to fluctuations in weather conditions, natural disasters (eg, drought, bushfires, floods, and cyclones), commodity and fuel prices, and currency exchange rates [1]. Remoteness increases the risk of mental illness, self-harm, and suicide [2]. Relative to urban areas, the suicide rate is 40% higher in rural areas, increasing to 100% higher in remote areas. Suicide rates in both rural and remote areas are also increasing faster than in capital cities (between 2012 and 2016, suicide rates increased by 9.2% outside capital cities, compared with 2% in capital cities) [3]. In particular, farmers are more likely to commit suicide than other occupations [4-6]. Those most at risk fit the following profile: most of them are men (>90%), are young (mean suicide age of 41 years), have recently separated or divorced (20%), live alone (33%), are more likely to be farm laborers than farm owners or managers, and have a precipitating mental condition [7].

### Barriers to Accessing Mental Health Services

Fewer mental health professionals work in rural and remote areas than in urban areas (70%-80% less than in major cities). Those living in rural communities may be reluctant to seek counseling services owing to stigma, community gossip, entrenched stoicism, and views that help seeking is a sign of weakness [8,9].

In recent decades, technological advances have led to the implementation of e-mental health services that can circumvent some of the barriers mentioned previously and make valuable contribution to service delivery in rural areas [10]. Various technology-based approaches have been developed, including text-based, audio-delivered, or even audio-visual counseling and web-based counseling services that offer a suite of delivery methods (eg, Talkspace [11,12]). Text-based counseling may be particularly well suited to individuals who are reluctant to seek help owing to stigma and stoicism [13]. Synchronous text-based counseling involves the simultaneous participation of 2 parties (eg, a client and a therapist), who engage in real-time communication (eg, via text-based messaging). Two main aspects of text-based counseling are likely to appeal to farmers living in rural and remote communities: (1) the anonymous nature of text-based interactions and (2) low-bandwidth delivery across great distances, which eliminates the need for transport or high levels of internet connectivity.

Evidence suggests that people generally disclose emotions in similar ways when using technology and face-to-face communication [14] and that text-based communication enables some people to better express their true-self qualities [15] or to disclose more personally confronting topics [16].

A consistent observation across studies is that text-based counseling takes longer than phone counseling [17,18] and generates fewer words than verbal exchanges [19]. Some patients expressed negative views about text-based communication related to less fluid interactions, reduced content covered, and impatience while waiting for the therapist to respond. Clients who prefer face-to-face or phone conversations have described text-based communication as too distant or impersonal. However, others have found the time delays created space to think, reflect, and communicate feelings without being disrupted by further questioning, as might occur in face-to-face sessions [20]. Those who viewed text-based counseling positively appreciated the distance, anonymity, security, privacy, and control over self-presentation [21].

### The Effectiveness of Text-Based Counseling

Text-based counseling has been shown to be as effective as traditional face-to-face counseling for a variety of conditions including depression [12,22], anxiety [12,23], and emotional problems [17,21]. Therapist-guided internet-delivered treatments are effective in treating a range of mental health conditions and can be as effective as face-to-face treatments [24-26].

Text-based delivery has been rated as better than or equal to face-to-face therapy in several dimensions, including convenience, effectiveness, making progress with problems, and having access to help when needed [11]. However, it should be noted that text-based counseling may not be the optimal mode of service delivery for all clients [18] and that those who do not engage during text-based therapy will not show clinical improvement [27,28].

### Computational Linguistic Analyses of the Text-Based Counseling

There is increasing evidence that analysis of e-mental health communications can be used to draw reliable inferences that can guide treatment. For instance, voice analysis systems have been developed that use artificial intelligence to improve treatment outcomes (eg, Eleos) or convey empathy and predict treatment engagement (eg, Lyssn AI). Text-based counseling is particularly attractive for subsequent computational linguistic analyses because it automatically documents the exchanges during the therapeutic process, thus avoiding the costs and difficulties associated with generating transcripts of audio-recorded sessions. This text chat offers possibilities that are not available to other service delivery methods: it permits the client and therapist to reread and reflect on their communication after the session has ended and it also opens up the research possibility of conducting analyses on text chats to identify linguistic patterns. Emerging literature suggests that language use patterns are reliable predictors of mental health status, but few studies have linguistically analyzed texts from individuals at risk of mental health problems. Most have mined social media [29-33] or web-based forums [34-37] to identify linguistic patterns that might be predictive of mental health status.

This study has been enabled by the development of computational linguistic tools, of which the most widely used is the Linguistic Inquiry and Word Count (LIWC) [38,39]. In addition to categorizing and counting words, LIWC offers an overview of the statistical distribution of words within predefined and psychologically meaningful categories. The capabilities of LIWC (and other similar programs or algorithms) have led researchers to explore the language use patterns of individuals with depression and other mental health conditions. Numerous studies have shown that increased use of first-person singular pronouns (eg, *I, me, my,* and *mine*) is indicative of depression [40-44], severity of depression and anxiety [45,46], general proneness to distress or negative emotionality [47,48], and suicidal ideation [49]. These promising findings suggest that language use patterns could conceivably serve as predictors of mental health, with potentially clinically significant applications.

Very few studies have analyzed text-based counseling. Nevertheless, the results from this small number of studies suggest that this may be a potentially fruitful avenue for future studies. Dirkse et al [50] found that greater use of negative emotion, anxiety, and sadness words positively correlated with heightened anxiety; greater use of negative emotion, sadness, and anger words positively correlated with heightened depression; and greater use of negative emotion and anger words positively correlated with heightened panic. Compatible findings showed that the use of negative emotion words predicted symptom improvement in outpatients being treated for personality disorders [51], and use of discrepancy words (eg, *would*, *should*, *wish*, and *hope*) reliably predicted depression improvement [52]. Patients with depression who used positive emotion words early in treatment tended to have good treatment outcomes, whereas the use of past focus words was associated with poor treatment outcomes [53]. These results suggest that word use may be used to determine an individual’s psychological condition and future prognosis.

Owen et al [54] examined word use in a support intervention for women with early-stage breast cancer. More frequent use of words expressing anxiety and sadness (but not anger) was significantly correlated with improved emotional well-being at follow-up, whereas greater expression of sadness (but not anxiety or anger) was associated with improved quality of life.

Although this is an emerging research area, the level of sophistication in the analyses is rapidly improving. Seabrook et al [55] were able to reliably predict depression severity using negative emotion word instability, and interestingly, they created an emoji and internet slang supplement to the LIWC dictionary, which increased the accuracy of depression identification. In addition, it may soon be possible to combine demographic, linguistic, behavioral, and social data to construct sophisticated models to identify at-risk individuals [56].

### This Study

This study examined an initiative funded by the Australian Government that provided text-based counseling to Australians in rural and remote communities through the *Virtual Psychologist* service [57]. The major aim of this study was to demonstrate the feasibility of using linguistic patterns in text-based counseling chats to predict whether an individual is experiencing depression, anxiety, or stress. The analysis was conducted using the LIWC software tool [38] on the text obtained from counseling sessions conducted over a 1-year period. The study also investigated whether language patterns were predictive of self-rated mental status. An additional consideration was to examine the characteristics of individuals who are using text-based counseling in rural and remote Australia.

## Methods

### Ethics Approval

This study was conducted in full compliance with the National Statement on Ethical Conduct in Human Research and approved by the Western Sydney University Human Ethics Committee (approval number H13309).

### Recruitment

Participants were 320 clients of the Virtual Psychologist text-based counseling service, who used the service between August 2019 and September 2020 for ≥1 sessions. Virtual Psychologist is a privately owned, for-profit organization that offers counseling over a range of platforms (eg, text, voice, or video). This study involved only those clients who engaged in live text-based counseling. On average, each session lasted for 52 (SD 16) minutes. All the therapists in the Virtual Psychologist service were qualified psychologists. Funded by the Australian government, the text-based counseling service has been provided free of charge to any Australian farmer who feels that they need such service. The Virtual Psychologist service has not been scientifically evaluated, although it uses evidence-based counseling approaches. The Virtual Psychologist service was advertised through various platforms, such as radio, television, and social media (eg, Twitter and Facebook). Some participants were also referred by friends, family members, volunteers, police, or physicians. Participants were able to have as many sessions as they wanted and could ask for a session when they felt they needed it. All sessions were initiated by the participant. In most instances, the participant ended the session, usually once they felt that their pressing concerns had been addressed. All participants lived in rural or remote communities across Australia. Data from participants aged <18 years were excluded from the study.

### Materials

*Virtual Psychologist* provided the data on a monthly basis to the researchers. The data consisted of the text from chat sessions, with metadata providing the date and time of each interaction and demographic information about the participant.

The LIWC tool [38] is the most widely used corpus of dictionaries for computational linguistic analyses of text data. It is a software program containing algorithms that enable it to count words belonging to different categories. To achieve this, LIWC compares words within an input text file with those within its dictionary. The output provides an overview of the statistical distribution of words within a text into predefined categories, including function words, pronouns, impersonal pronouns, verbs, auxiliary verbs, and past-tense words.

The LIWC dictionaries were customized to suit the Australian data set. To achieve this, Australian spellings were added to the standard LIWC American spellings (eg, Australian *agonise vs* US *agonize*), and, where necessary, equivalent Australian words were also added (eg, Australian *mobile vs* US *cellphone*). The Australianized dictionary is described in Multimedia Appendix 1.

### Procedure

Participants were provided with the service’s terms and conditions at the first point of contact with the *Virtual Psychologist* service. Then, they completed a short demographic survey, which was deidentified. Text from counseling sessions between participants and therapists were also deidentified (ie, names of people, workplaces, and landmarks were removed). Immediately after each session, participants received an SMS with a link to a short client survey regarding their mental health presenting problems (choice from a list of 20 common presenting problems; Table 1) and their experience with the *Virtual Psychologist* service. The survey also contained the single-item self-rating of mental health, “How would you rate your mental health now?*”* (adapted from the study by Althoff et al [58]) on a 5-point scale ranging from *poor* to *excellent*. The survey was optional for participants, and the response rate for the self-rating of mental health was found to be relatively low. In July 2020, the participants who had not completed the survey in their sessions were contacted and asked to respond to the self-rating.

**Table 1:** Distribution of the number of text-based counseling sessions completed by each of the 270 participants. A total of 94.8% (256/270) of participants completed between 1 and 7 sessions (N=270).

Number of sessions	Participants, n (%)
1	98 (36.3)
2	61 (22.6)
3	32 (11.9)
4	34 (12.6)
5	15 (5.6)
6	12 (4.4)
7	4 (1.5)
8	3 (1.1)
9	3 (1.1)
10	2 (0.7)
11	1 (0.4)
12	2 (0.7)
13	2 (0.7)
14	1 (0.4)

### Data Processing

**Figure 1:**
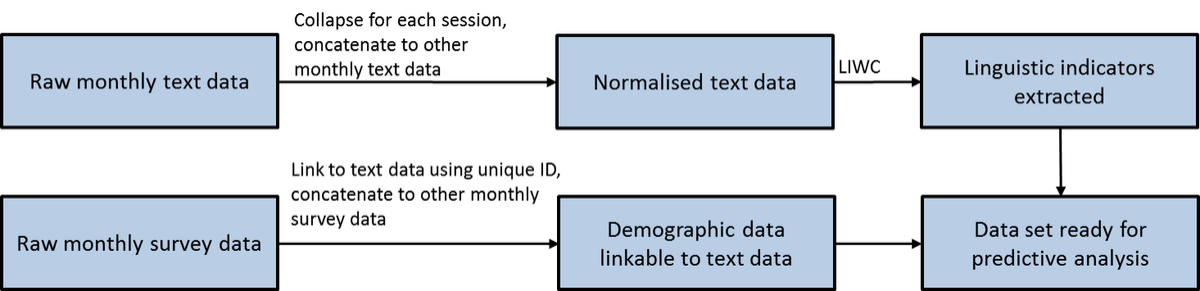
Workflow for the data processing and analyses. LIWC: Linguistic Inquiry and Word Count.

Owing to privacy reasons, names, locations, and other identifying information in the raw text data were manually identified and replaced using labels such as NAME or PLACE by Virtual Psychologist before the data were provided to the research team. Each participant and chat session were assigned a unique participant ID and session ID, respectively. For each session, the chat text from the participant was first aggregated. To ensure compatibility with LIWC, the aggregated text data were preprocessed (normalized and restructured) using the MATLAB software developed by MathWorks. Normalization involves common text cleaning operations, including removing punctuations, extra spaces, and returns and converting all words to lowercase. Emojis and other internet slangs (if any) were left unchanged, together with spelling errors. Chat sessions containing <30 words from participants were removed; this was usually owing to participants being unable to continue soon after initiating the session (eg, poor cellular network coverage or work-related or personal situation requiring them to leave the session). A total of 33.02% (381/1154) of the sessions for 15.6% (50/320) of the participants met this criterion and were excluded from the analysis. The final data set comprised 66.98% (773/1154) of the sessions, involving 84.4% (270/320) of the participants. Figure 1 shows the workflow for data processing and analyses.

### Data Analysis

Linguistic indicators were extracted for each session using LIWC [38,39]. We explored the relationships between the linguistic patterns in the chat sessions and participants’ self-reported psychological concerns at service entry and their mental well-being self-rating after receiving counseling. Of all possible linguistic indicators, the following 21 indicators were selected as independent variables (predictors) to be used in the predictive analysis: word count, analytical thinking score, clout score, authenticity score, emotional tone, first-person singular pronouns, positive emotions, negative emotions, causation, insight, discrepancy, social processes, functional words, other words, affect, cognitive processes, drives, personal concerns, past focus, present focus, and future focus. The selection of these 21 indicators was based on the following reasons: first, they cover most of the word categories available in LIWC; second, the rest of the indicators available in LIWC, such as punctuation marks and relativity (motion, space, and time), were considered to have little relevance to self-reported presenting problems and self-rated mental well-being, and thus were excluded; and third, previous studies [40-54] showed that some mental health problems, such as anxiety and depression, were highly correlated with a selection of these indicators. Participants’ self-reported mental health problems at service initiation and self-rated mental well-being from the survey were treated as dependent variables in the predictive analysis. Quadratic discriminant analyses with 5-fold cross validation were conducted in MATLAB to explore the relationship between the independent and dependent variables. Each discriminant model uses a different combination of the 21 linguistic indicators as predictors to calculate the probabilities of classification response and, then, outputs the predicted classification label based on the highest probability. The performance of each model was compared to determine which discriminant model performed optimally using 3 metrics: Area under the Receiver Operating Characteristic curve (AUC), general prediction accuracy, and average prediction accuracy when the prediction probability was set to 70%, 80%, and 90%. Models with highest AUC and accuracy and lowest number of predictors were preferred.

## Results

### Sample Description

The text data used in the analyses reported here were collected between August 2019 and September 2020. They consist of 100% (1154/1154) of the text-based counseling sessions from 100% (320/320) of the participants who engaged with the *Virtual Psychologist* service. Following data cleaning and preprocessing, the final data set for linguistic and predictive analysis comprised 66.98% (773/1154) of the sessions from 84.4% (270/320) of the participants. The distribution of sessions per participant is shown in Table 1.

### Characteristics of Individuals Who Engaged in Text-Based Counseling

On average, participants completed 3.6 (SD 3.2) sessions of text-based counseling; however, there was considerable variability in the number of sessions completed. Most participants (256/270, 94.8%) engaged in 1 to 7 sessions; however, some engaged in as many as 14 sessions. Approximately one-third (98/270, 36.3%) of the participants engaged in only 1 session. For each session, the client sent an average of 11 (SD 11; range 1-84) messages. The total number of words per session also varied widely, with an average session containing 357 (SD 300) words exchanged between the therapist and the client. This is consistent with the literature reporting that text-based chat is slower than verbal communication and results in fewer words being exchanged between conversational partners [17,18].

Data collection commenced in August 2019. However, as shown in Table 2, the number of monthly sessions increased from March 2020 owing to increase in the number of participants using the counseling service. This was likely precipitated by 2 events. First, Australia experienced unprecedented bushfires in late 2019, extending into early 2020, and rural areas were the most badly affected. Second, the first confirmed case of COVID-19 in Australia was identified on January 25, 2020 [59], resulting in Australian borders being closed to nonresidents on March 20, 2020, and government restrictions (social distancing rules and closing of nonessential services) being put in place on March 21, 2020. In our text-based chat data, bushfire-related events were mentioned 1 to 9 times per month, and COVID-19 was mentioned 2 to 22 times per month during the period from December 2019 to September 2020.

Regarding sex, most participants were women (167/270, 61.9%). As shown in Table 3, women outnumbered men by 3:1 and also completed more sessions. Of the 270 participants, 44 (16.3%) participants did not disclose their sex.

The age distribution of the participants is shown in Table 4. The sample primarily comprised young adults (age ranges 18-21 years and 22-29 years). This is consistent with reports that individuals who are comfortable with using technology are more likely to engage in text-based counseling. However, interestingly, the discrepancy between participants aged 18 to 21 years and 30 to 40 years decreases when we inspect the number of sessions completed, suggesting that the average participants aged 30 to 40 years engaged in a greater number of counselling sessions than the average participants aged 18 to 21 years. This is encouraging, as men aged 41 years are most at risk of serious mental health problems and suicide [60]. Older adults were fewer in number and, on average, engaged in fewer sessions than their younger counterparts (2 sessions).

**Table 2:** Number of text-based counseling sessions completed by participants in each month from August 2019 to September 2020 (N=773).

Month and year	Sessions, n (%)
August 2019	45 (5.8)
September 2019	39 (5)
October 2019	33 (4.3)
November 2019	46 (5.9)
December 2019	43 (5.6)
January 2020	26 (3.4)
February 2020	18 (2.3)
March 2020	54 (6.9)
April 2020	99 (12.8)
May 2020	76 (9.8)
June 2020	56 (7.2)
July 2020	73 (9.4)
August 2020	97 (12.5)
September 2020	68 (8.8)

**Table 3:** Number of sessions and number of participants for each sex.

Sex	Sessions (N=773), n (%)	Participants (N=270), n (%)
Female	500 (64.7)	167 (61.9)
Male	162 (21)	59 (21.9)
Undisclosed	111 (14.3)	44 (16.3)

**Table 4:** Number of sessions completed by each age category and participants’ age distribution.

Age categories (years)	Sessions (N=773), n (%)	Participants (N=270), n (%)
18-21	196 (25.4)	74 (27.4)
22-29	275 (35.6)	77 (28.5)
30-40	133 17.2)	43 (15.9)
41-50	78 (10.1)	39 (14.4)
51-60	36 (4.7)	15 (5.6)
61-70	29 (3.8)	12 (4.4)
Undisclosed	26 (3.4)	10 (3.7)

Upon referral to the *Virtual Psychologist* counseling service, each participant’s self-reported mental health concern was recorded (Table 5). The number of presenting problems reported by each participant ranged from 1 to 5, with an average value of 1.76 (SD 0.96). The top 3 mental health conditions that clients presented with were anxiety, depression, and stress, and they comprised approximately half of the total number of sessions. These 3 presenting problems sometimes overlapped for the same individual. Of the 270 participants, 26 (9.6%) participants reported having both depression and anxiety, 10 (3.7%) reported both depression and stress, 9 (3.3%) reported both anxiety and stress, and 4 (1.5%) reported having all the 3 problems. Approximately one-fourth of all sessions fell into the *other* and *undisclosed* categories. Apart from the explanation that participants may not be able to find the right category for their problems, this could also suggest that even for an anonymous and privacy-focused method of e-mental health service provision, there remains a considerable number of individuals for whom disclosure, and presumably stigma, remains as an issue (even when it does not prevent seeking help).

Of the 773 completed sessions, 165 (21.3%) responses obtained from 38.9% (105/270) of the participants were recorded for the single-item self-rating of mental well-being, “How would you rate your mental health now?*”* on a 5-point scale ranging from *poor* to *excellent*. A total of 29.7% (49/165) of responses from 40.9% (43/105) of the participants were collected immediately after the counseling sessions, and the remaining responses were collected in July 2020. Of the 21.3% (165/773) sessions with responses to the self-rating question, 51.5% (85/165) reported anxiety, depression, and stress as presenting problems, whereas for those without responses to the self-rating question, 44.7% (272/608) reported these 3 presenting problems. Among those participants who responded to the self-rating question, 72.4% (76/105) were women, 15.2% (16/105) were men, and 12.4% (13/105) chose to remain undisclosed, whereas among those who did not respond, 57.6% (95/165) were women, 21.8% (36/165) were men, and 20.6% (34/165) chose to remain undisclosed. Regarding age distribution, of the participants who responded to the self-rating question, those in the age groups of 18 to 21 years, 21 to 29 years, and 30 to 40 years were 4% less than those who did not respond, but those in the age groups of 41 to 50 years, 51 to 60 years, and 61 to 70 years were 5.5% more than those who responded. Therefore, older women who reported anxiety, depression, and stress were more likely to respond to the self-rating question. As shown in Table 6, most participants chose *fair*, although responses varied widely, and made use of the full range of response options available. The average self-rating score was 2.7 (SD 1.3).

**Table 5:** Presenting problems that led participants to seek counseling, expressed as distribution of the number of text-based counseling sessions completed^a^.

Presenting problem	Sessions (N=773), n (%)	Participants (N=270), n (%)
Anxiety	152 (19.7)	71 (26.3)
Depression	143 (18.5)	79 (29.3)
Stress	61 (7.9)	34 (12.6)
Family issues	50 (6.5)	30 (11.1)
Relationship issues	49 (6.3)	34 (12.6)
Grief and loss	25 (3.2)	12 (4.4)
Trauma issues	15 (1.9)	11 (4.1)
Suicidal thoughts	13 (1.7)	10 (3.7)
Anger	6 (0.8)	6 (2.2)
Work problems	6 (0.8)	5 (1.9)
Domestic violence	4 (0.5)	4 (1.5)
Isolation or loneliness	4 (0.5)	2 (0.7)
Critical incident	3 (0.4)	3 (1.1)
Self-harm	3 (0.4)	3 (1.1)
COVID-19	2 (0.2)	2 (0.7)
Eating disorders	1 (0.1)	1 (0.4)
Friend issues	1 (0.1)	1 (0.4)
Health concerns	1 (0.1)	1 (0.4)
LGBTI^b^ issues	1 (0.1)	1 (0.4)
Physical abuse	1 (0.1)	1 (0.4)

^a^Technical issues and undisclosed presenting problems (232/773, 30% of the sessions for 121/270, 44.8% of the participants) are not listed.

^b^LGBTI: lesbian, gay, bisexual, transgender, and intersex.

**Table 6:** Participant responses to the single-item self-rating of mental well-being, “How would you rate your mental health now?” on a 5-point scale ranging from poor to excellent (N=165).

Self-ratings of mental well-being	Sessions, n (%)
Poor	34 (20.6)
Fair	53 (32.1)
Good	30 (18.2)
Very good	29 (17.6)
Excellent	19 (11.5)

### Linguistic Analysis

The 4 basic LIWC scores are shown in Table 7: analytical thinking, clout, authenticity, and emotional tone.

**Table 7:** Descriptive statistics of Linguistic Inquiry and Word Count scores for the basic summary variables: analytical thinking, clout, authenticity, and emotional tone. Scores are calculated based on the text from each session. Summary variable scores range from 1 to 99.

Indicators	Score, mean (SD)	Score, median (range)
Analytical thinking	24 (19)	19 (1-95)
Clout	34 (26)	28 (1-99)
Authenticity	75 (26)	86 (1-99)
Emotional tone	57 (34)	60 (1-99)

The distributions of these 4 basic LIWC dimensions are shown in Table 8. Analytical thinking scores showed a shallow positive skew, with most of those scores falling within the range of 0 to 50. This indicates that participants were using a language style similar to a narrative, focused on their personal experiences. High analytical thinking scores are associated with better academic performance in tertiary education [61]. The observed concentration of scores on the other half of the scale appears to be a valid representation of the sample population being studied, that is, farmers living in rural areas.

Clout refers to social status, confidence, or leadership [62]. Clout scores showed a shallow positive distribution and were somewhat more evenly distributed across the range of scores. This may reflect different ranks or responsibilities within the sample, such as farm laborers versus farm managers and owners.

Authenticity scores showed a sharp negative distribution. Higher authenticity scores indicate truthfulness, humility, and vulnerability [63,64]. Encouragingly, this indicates that most participants were using language associated with being truthful. This is consistent with literature suggesting that text-based counseling offers high degree of privacy and anonymity, giving users time and space to select the right words to express themselves [20,21] and reveal more truthful information [16].

Emotional tone scores were the most evenly distributed among the 4 LIWC summary variables. High scores (>50) reflect more positive emotional tone [65]. Participants spanned the full range of the scale, and the mean was 57, indicating a neutral to positive emotional tone. There was a gradual increase in the number of scores toward the negative end of the scale, indicating that the sample contained individuals experiencing severe negative emotions. Encouragingly, there was also a sharp spike in the most positive interval of the scale (90-99), indicating that many participants were using positive emotion words, which included positive feelings or expressions of gratitude to the therapist.

**Table 8:** Distribution of Linguistic Inquiry and Word Count scores for the basic dimensions—analytical thinking, clout, authenticity, and emotional tone (N=773).

Score (range)	Analytical thinking, n (%)	Clout, n (%)	Authenticity, n (%)	Emotional tone, n (%)
0-10	180 (23.3)	134 (17.3)	23 (3)	86 (11.1)
11-20	231 (29.9)	165 (21.3)	21 (2.7)	64 (8.3)
21-30	142 (18.4)	114 (14.7)	32 (4.1)	72 (9.3)
31-40	84 (10.9)	93 (12)	26 (3.4)	57 (7.4)
41-50	50 (6.5)	59 (7.6)	40 (5.2)	55 (7.1)
51-60	32 (4.1)	68 (8.8)	37 (4.8)	53 (6.8)
61-70	24 (3.1)	45 (5.8)	60 (7.8)	50 (6.5)
71-80	18 (2.3)	32 (4.1)	86 (11.1)	73 (9.4)
81-90	10 (1.3)	24 (3.1)	132 (17.1)	61 (6.6)
91-100	2 (0.2)	39 (5)	316(40.9)	212 (27.4)

The category with the best-established relationship to mental health is that of first-person singular pronouns. Overuse of first-person singular pronouns (eg, *I, me, my,* and *mine*) is a marker of depression [40,42,44] and predicts the severity of depressive symptoms 8 months after treatment [46]; however, recent findings suggest that first-person singular pronoun use may be indicative of general proneness to distress or negative emotions rather than of depression specifically [47,48]. As shown in Table 9, use of first-person singular pronouns comprised 10% of the words.

Social process words (eg, *share* and *we*) also comprised approximately 10% of the words within a session. This is to be expected, as the participants were reflecting on their relationships with others.

Use of positive (eg, *happy* and *brave*) and negative (eg, *sad* and *desperate*) emotion words is known to relate to mental health and symptom severity. Individuals with depression use more negative and fewer positive emotion words [41,43]. Reduced use of negative emotion words predicts symptom improvement [51]. In this study, the frequency of positive emotion words shows a positive skew, as may be expected for individuals undergoing psychological counseling.

**Table 9:** Percentage of words falling within the Linguistic Inquiry and Word Count categories: first-person singular pronouns, positive emotion, negative emotion, causation, discrepancy, insight, and social processes. The indicators are calculated based on the text from each session.

Indicators	Words (%), mean (SD)	Words (%), median (range)
First-person singular pronouns	10 (3.3)	10.3 (0-22.5)
Positive emotion	5.3 (3.2)	4.4 (0-27.8)
Negative emotion	2.7 (1.7)	2.6 (0-10.1)
Causation	1.6 (1.1)	1.6 (0-6.5)
Insight	2.8 (1.7)	2.8 (0-10.3)
Discrepancy	2 (1.5)	1.8 (0-12.5)
Social processes	10.2 (4.9)	9.5 (0-35.3)

There is some evidence that the use of absolutist words (ie, words that indicate certainty such as *always*, *totally*, *constantly*, *forever*, *completely*, and *entire*) may predict suicidal ideation better than negative emotion words or first-person pronouns [34,36]. We expect those participants experiencing greater psychological distress to make greater use of causation words [50,51] and less use of discrepancy words [52]. As shown in Table 10, the distributions of causation words (eg, *because*, *aggravate*, and *basis*) resemble that of the negative emotion and insight categories. The distribution of discrepancy words (eg, *would not*, *unusual*, *abnormal,* and *impossible*) shown in Table 10, is moderately more positively skewed, which suggests that there is considerable variability within the data regarding the severity of mental health issues being experienced by participants.

**Table 10:** Distribution of words per session for the Linguistic Inquiry and Word Count categories—first-person singular pronouns, positive emotion words, negative emotion words, causation words, insight words, discrepancy words, and social processes.

Indicators	Words (%), range; %									
First-person singular pronouns	0-2.3; 1.9	2.3-4.6; 4.9	4.6-6.9; 10.5	6.9-9.2; 19.4	9.2-11.5; 29.1	11.5-13.8; 24.5	13.8-16.1; 8.3	16.1-18.4; 1	18.4-20.7; 0.3	20.7-23; 0.1
Positive emotion	0-2.8; 16	2.8-5.6; 51	5.6-8.4; 19.7	8.4-11.2; 7.6	11.2-14; 3.6	14-16.8; 1.4	16.8-19.6; 0.1	19.6-22.4; 0.3	22.4-25.2; 0.1	25.2-28; 0.1
Negative emotion	0-1.1; 14.9	1.1-2.2; 23.5	2.2-3.3; 28.8	3.3-4.4; 17.6	4.4-5.5; 8.5	5.5-6.6; 4.4	6.6-7.7; 1.8	7.7-8.8; 0.1	8.8-9.9; 0	9.9-11; 0.3
Causation	0-0.65; 21	0.65-1.3; 15.4	1.3-1.95; 27.8	1.95-2.6; 21.2	2.6-3.25; 9.8	3.25-3.9; 2.7	3.9-4.55; 0.9	4.55-5.2; 0.5	5.2-5.85; 0.3	5.85-6.5; 0.4
Insight	0-1.1; 16.2	1.1-2.2; 20.6	2.2-3.3; 27.2	3.3-4.4; 21.3	4.4-5.5; 9.3	5.5-6.6; 4	6.6-7.7; 0.9	7.7-8.8; 0.1	8.8-9.9; 0.3	9.9-11; 0.1
Discrepancy	0-1.3; 29.2	1.3-2.6; 45	2.6-3.9; 17.2	3.9-5.2; 4.8	5.2-6.5; 1.8	6.5-7.8; 1.2	7.8-9.1; 0.5	9.1-10.4; 0.1	10.4-11.7; 0	11.7-13; 0.1
Social processes	0-3.6; 4.9	3.6-7.2; 23.4	7.2-10.8; 30.7	10.8-14.4; 23	14.4-18; 11.3	18-21.6; 4.4	21.6-25.2; 1.6	25.2-28.8; 0.3	28.8-32.4; 0.4	32.4-36; 0.1

### Predictive Analysis

#### Overview

**Figure 2:**
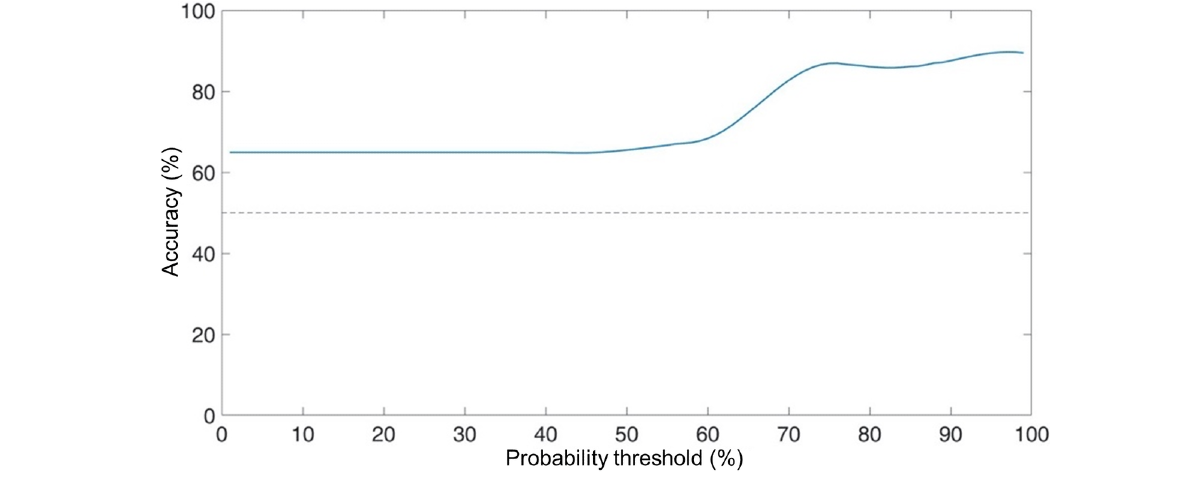
Example of an accuracy curve showing prediction accuracy when the prediction probability is set at different thresholds. The dashed line shows the accuracy at chance level (50% for binary classification).

Quadratic discriminant analyses were conducted to explore the relationship between the linguistic categories provided by LIWC and mental health status. Each discriminant model used a different combination of linguistic categories as predictors to calculate the probabilities of classification response and, then, outputs the predicted classification label based on highest probability. Then, 5-fold cross validation was applied to each of the discriminant models. The performance of the different models was compared to determine which discriminant model performed optimally. Model performance was assessed using the following three metrics:

1. Examining the AUC: Interpretation of AUC varies across disciplines. In applied psychology, given the large number of variables that can influence human behavior, AUC values ≥0.71 are considered as strong effects [66].
2. General prediction accuracy.
3. Average prediction accuracy when the prediction probability was set to 70%, 80%, and 90% (calculated from the accuracy curve; refer to example shown in Figure 2). The prediction accuracy increases considerably when the prediction probability is >70%.

#### Binary Classification of Mental Health Presenting Problem

First, we examined whether language use patterns could be used to discriminate the top 3 presenting problems (ie, anxiety, depression, and stress; Table 5) from the remaining pool of presenting problems (binary classification). This distinction was deemed important because these 3 presenting problems were the most frequently occurring problems within the data set, and they are the most studied in the literature. Accurate, scalable classification would be useful for screening and for targeted interventions.

**Figure 3:**
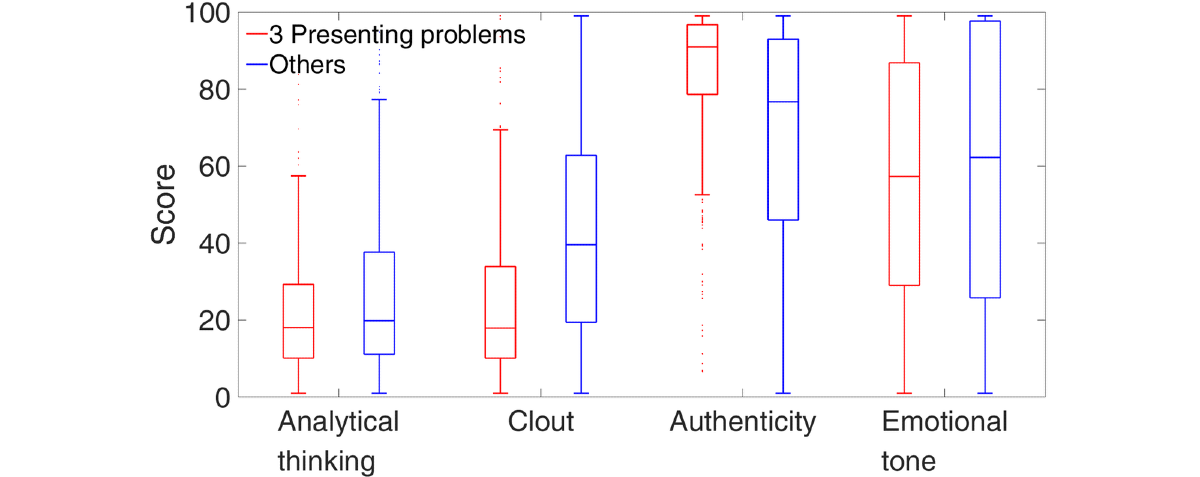
Boxplots of the 4 basic Linguistic Inquiry and Word Count counts (clout, authenticity, emotional tone, and analytical thinking) for the top 3 presenting problems (red) and the remaining pool of other presenting problems (blue).

Before reporting the binary classification results, we inspect the differences in LIWC counts between the top 3 presenting problems and the pool of remaining presenting problems. To do this, we present boxplots for each LIWC count of interest. Boxplots are a standardized way of visualizing the distribution of data by presenting the median, first and third quartiles (edges of the box), and minimum and maximum (error bars); they indicate the spread of the data, whether it is symmetrical, how tightly it is grouped, and skewness. Differences in boxplots between classification options would be indicative of accurate predictions in the corresponding discriminant analyses. Figure 3 shows boxplots for the 4 basic LIWC counts. The top 3 presenting problems, relative to the others, were differentiated by lower clout and authenticity scores and higher emotional tone scores.

**Figure 4:**
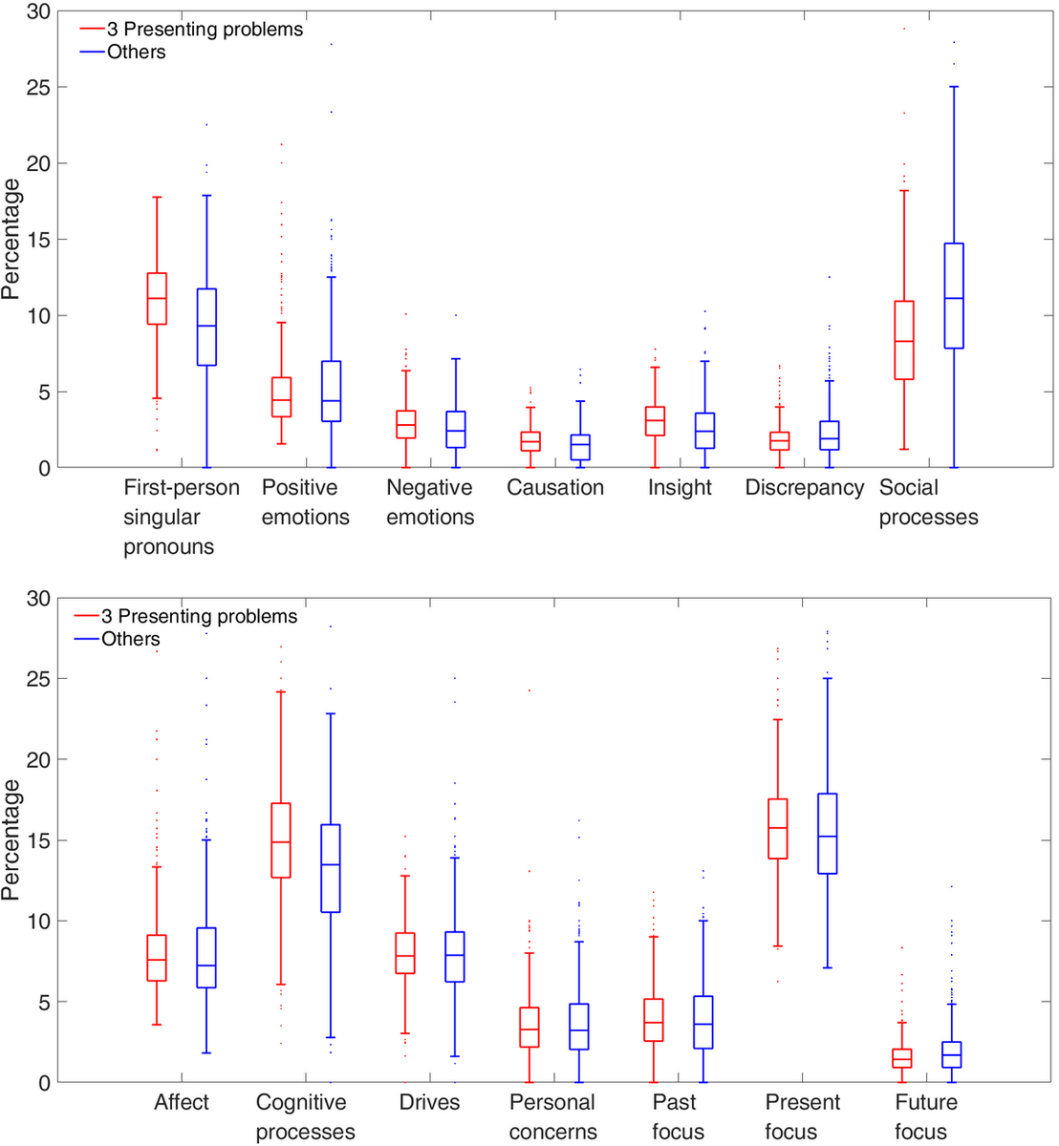
Boxplots of the Linguistic Inquiry and Word Count categories for the top 3 presenting problems (red) and the remaining pool of other presenting problems (blue).

Figure 4 shows boxplots for the LIWC categories. In the upper panel, the top 3 presenting problems were differentiated from the others by an increased use of first-person singular pronouns and insight words, but less use of discrepancy and social process words. In the lower panel, the 2 classifications may also be differentiated by words in 3 predefined LIWC categories: *cognitive processes*, *future focus*, and *drives*.

Table 11 presents the evaluation metrics (AUC, general accuracy, and average accuracy) for the best 5 discriminant models for discriminating the top 3 from the remaining pool of presenting problems using combinations of 1 to 5 and all predictors (binary classification). The data used in the binary classification comprised linguistic predictors extracted from 66.98% (773/1154) of the sessions with 84.4% (270/320) of the participants. Table 11 shows that general accuracy of the best discriminant models reached approximately 70% (exceeding the 50% chance level), with AUC of 0.76, indicating good discrimination and a strong effect. When the confidence of prediction was high, the average accuracy of prediction reached approximately 80%. Interestingly, increasing the number of predictors in the discriminant models did not offer continuous improvements in any of the evaluation metrics. Regarding the trade-off between model performance (here, accuracy and AUC) and model size (ie, the number of predictors used), models with 3 to 4 predictors seem to be optimal in terms of the balance between good accuracy and model complexity. Regarding LIWC categories, clout, future focus, discrepancy, emotional tones, drives, social processes, insight, and first-person singular pronouns were the most frequently occurring predictors among the discriminant models listed in Table 11.

**Table 11:** Best 5 models for discriminating the top 3 from the remaining pool of presenting problems.

Number of predictors and predictor names			AUC^a^		General accuracy (%)		Average accuracy^b^ (%)		F1 score
**1**									
	Clout score	0.70		64.9		86		0.68	
	Social processes	0.68		62.2		84.2		0.64	
	Authenticity score	0.66		61.4		81.5		0.67	
	First-person singular pronouns	0.66		62.7		84		0.65	
	Word count	0.64		56.8		N/A^c^		0.45	
**2**									
	Clout score+discrepancy	0.74		66.9		80.4		0.70	
	Clout score+functional	0.73		67.3		82.5		0.70	
	Clout score+future focus	0.73		67		81		0.70	
	Clout score+drives	0.73		66		82.9		0.68	
	Clout score+insight	0.73		68.2		83.6		0.69	
**3**									
	Clout score+discrepancy+focus future	0.75		67.1		77.6		0.70	
	Clout score+drives+future focus	0.75		67.8		79.4		0.70	
	Insight+social processes+functional	0.74		67.8		81.8		0.69	
	Clout score+authenticity score+future focus	0.74		67		78		0.69	
	Clout score+insight+drives	0.74		68.3		81.5		0.70	
**4**									
	Clout score+positive emotions+discrepancy+future focus	0.76		67.9		77.7		0.71	
	Clout score+emotional tone score+discrepancy+future focus	0.76		66.9		78.6		0.70	
	First-person singular pronouns+discrepancy+social processes+future focus	0.75		67.7		77.4		0.70	
	Clout+first-person singular pronouns+discrepancy+future focus	0.75		68.3		77.8		0.71	
	Clout+insight+drives+future focus	0.75		68.4		79.1		0.70	
**5**									
	Clout score+emotional tone score+discrepancy+functional+future focus	0.76		68.8		76.5		0.70	
	Clout score+emotional tone score+discrepancy+drives+future focus	0.76		67		77.3		0.71	
	Clout score+emotional tone score+discrepancy+social processes+future focus	0.76		68.4		77.9		0.71	
	Clout score+emotional tone score+insight+discrepancy+future focus	0.76		68.3		77.8		0.71	
	Word count+clout score+emotional tone score+discrepancy+ffuture focus	0.76		67.7		78.5		0.70	
21^d^	All predictors	0.72		63.8		67.9		0.66	

^a^AUC: Area under the Receiver Operating Characteristic curve.

^b^Average accuracy when the predicted probability threshold is set to 70%, 80%, and 90%.

^c^N/A: not applicable.

^d^The best models with 6-20 predictors (total of 75 items) have lower AUC, general accuracy, average accuracy, and F1 score, thus are omitted.

#### Multiclass Classification of Presenting Problem

**Figure 5:**
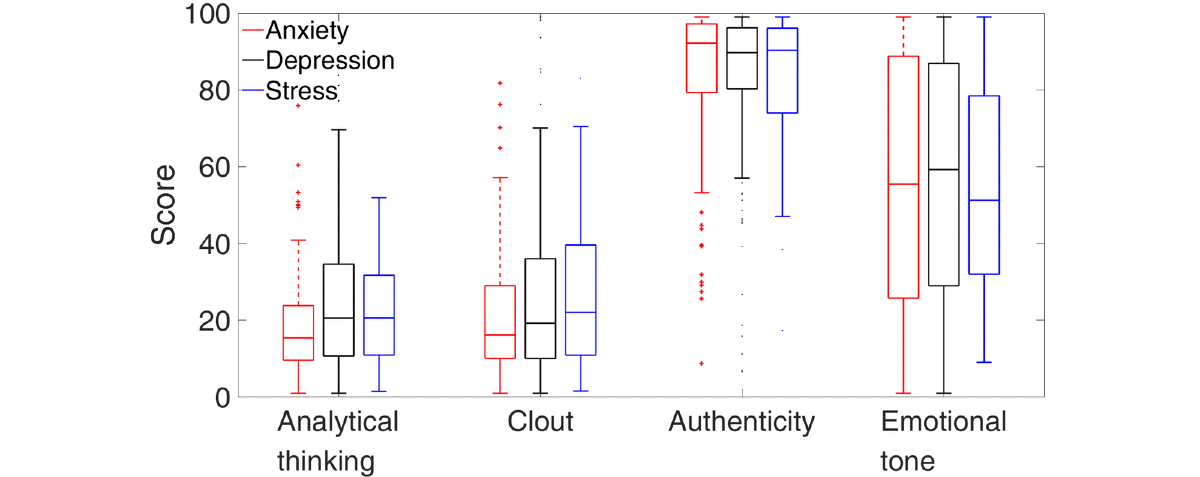
Boxplots of the 4 basic Linguistic Inquiry and Word Counts (analytical thinking, clout, authenticity, and emotional tone) for the top 3 presenting problems: anxiety (red), depression (black), and stress (blue).

The second set of results relates to the task of using language patterns to differentiate between the top 3 mental health presenting problems (ie, anxiety, depression, or stress). For this multiclass classification, chance level is 33.3%. Figure 5 shows boxplots for the 4 basic LIWC counts. For all the 4 counts, there was considerable overlap across the 3 presenting problems. Of the 4 counts, analytical thinking score was the most promising for differentiating anxiety from depression and stress (red is lower than black and blue).

**Figure 6:**
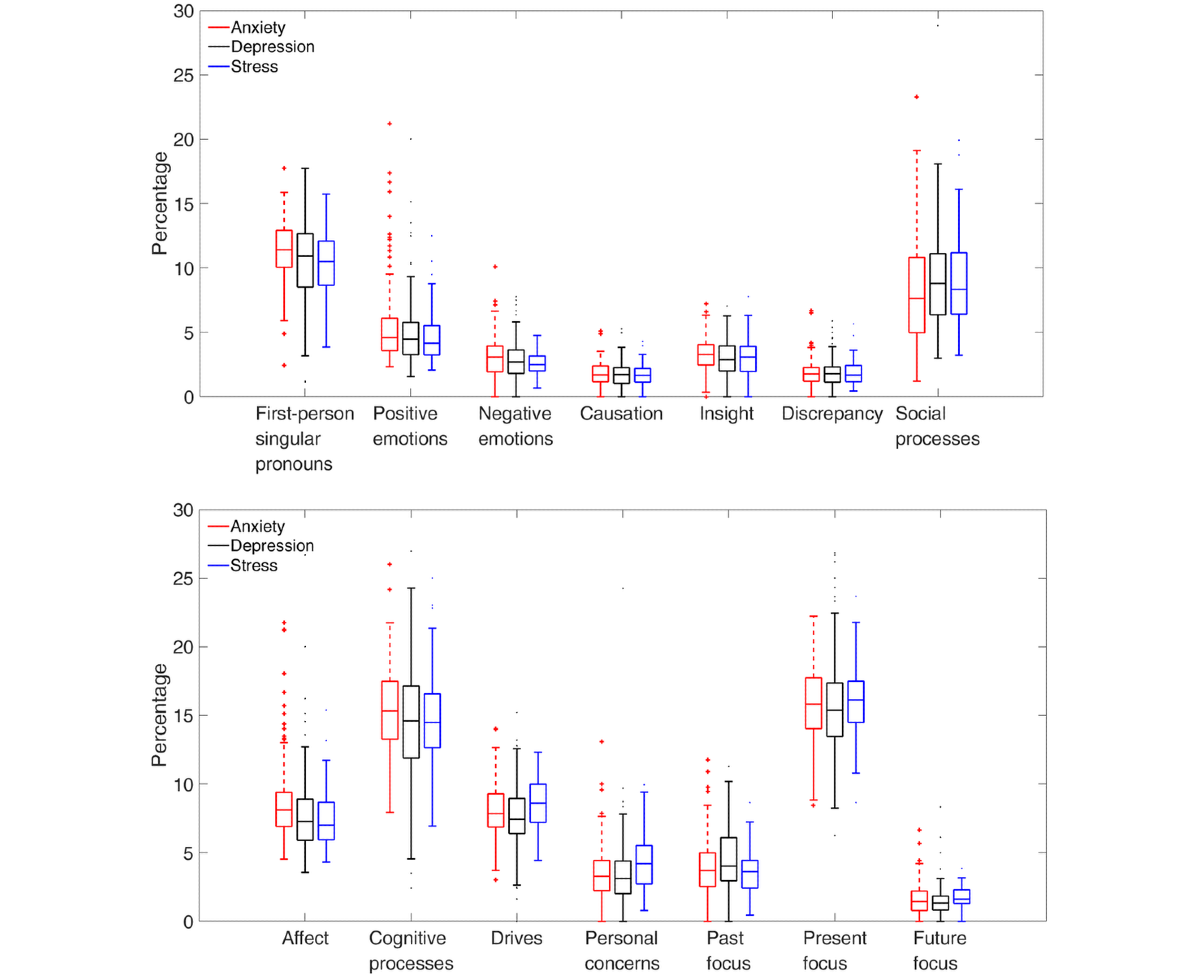
Boxplots of the Linguistic Inquiry and Word Count categories for each of the top 3 presenting problems: anxiety (red), depression (black), and stress (blue).

Figure 6 shows individual boxplots for the LIWC categories for each of the top 3 presenting problems. Again, there was considerable overlap across the top 3 presenting problems, suggesting that they share common features. Of the available LIWC categories, the most promising category in terms of differentiation was the first-person singular pronouns, which showed elevated counts for anxiety and depression relative to stress. However, the high degree of overlap across the LIWC categories suggests that it may be difficult to differentiate the top 3 presenting problems from one another.

Table 12 presents the evaluation metrics for the best 5 discriminant models for discriminating between the top 3 mental health presenting problems (anxiety, depression, and stress) using combinations of 1 to 5 and all predictors. The data used in the classification comprised linguistic predictors extracted from 30.85% (356/1154) of the sessions with 44.7% (143/320) of the participants. Table 12 shows that the general accuracy of the best discriminant models was slightly >50% (compared with 33.3% chance level). Cohen κ coefficient reached 0.21, showing fair agreement between prediction and ground truth. When the confidence of prediction was high, the average accuracy of prediction for most discriminant models were between 50% and 70%. Increasing the number of predictors did not substantially improve prediction accuracy. These results suggest that it is difficult to differentiate the top 3 presenting problems (ie, anxiety, depression, and stress) on the basis of LIWC categories, even though performance was well above chance level. Of the LIWC categories, analytical thinking score, cognitive processes, first-person singular pronouns, past focus, and present focus were the most frequently occurring predictors among the discriminant models listed in Table 12.

**Table 12:** Best 5 discriminant models for differentiating between the top 3 mental health presenting problems (anxiety, depression, and stress).

Number of predictors and predictor names			Cohen κ coefficient		General accuracy (%)		Average accuracy (%)
**1**							
	Analytical thinking score	0.12		49.2		71.6	
	Cognitive processes	0.12		49.2		52.6	
	Affect	0.12		48.6		33.3	
	First-person singular pronouns	0.09		47.2		83.3	
	Social processes	0.09		46.9		N/A^a^	
**2**							
	Analytic thinking score+first-person singular pronouns	0.14		50.3		75.1	
	Analytical thinking score+past focus	0.14		50.3		74.2	
	Analytical thinking score+cognitive processes	0.14		50		70.6	
	First-person singular pronouns+cognitive processes	0.14		50		54.2	
	First-person singular pronouns+past focus	0.14		50		57.1	
**3**							
	First-person singular pronouns+negative emotions+functional	0.18		50.6		66.8	
	Analytical thinking score+past focus+present focus	0.17		52		71.7	
	Analytical thinking score+emotional tone score+drives	0.17		51.4		77.5	
	Emotional tone score+cognitive processes+drives	0.17		51.4		66.7	
	First-person singular pronouns+cognitive processes+present focus	0.17		51.4		56.2	
**4**							
	Negative emotions+social processes+functional+focus present	0.21		50.8		68.7	
	Negative emotions+affect+drives+past focus	0.21		48.9		60.6	
	Analytical thinking score+affect+cognitive processes+present focus	0.2		52		64.6	
	Word count+analytical thinking score+causation+past focus	0.19		52		66.1	
	Word count+analytical thinking score+causation+present focus	0.19		52		62.7	
21^b^	All predictors	0.09		43.3		46.3	

^a^N/A: not applicable.

^b^The best models with 5-20 predictors (total of 80 items) have lower Cohen κ coefficient, general accuracy, and average accuracy, thus are omitted.

#### Binary Classification of Self-rating of Mental Well-being

The next set of analyses was regarding whether language use patterns can identify individuals with the poorest future mental health status by discriminating those individuals who rated their health as *poor* from the rest, that is, those who assigned the ratings *fair* to *excellent* (binary classification). Again, this distinction is important because those participants who rated their mental health as *poor* are more likely to require targeted intervention.

Discriminant models used all participants (105/105, 100%) and linguistic indicators extracted from 21.3% (165/773) of the corresponding chat sessions. Table 13 presents the evaluation metrics for the best 5 discriminant models for discriminating *poor* rating for self-rated mental health from other ratings ranging from *fair* to *excellent* using combinations of 1 to 5 and all predictors in each model. Table 13 shows that the general accuracy of the best discriminant models reached approximately 80% (exceeding the 50% chance level), with AUC reaching 0.73, showing good discrimination. When the confidence of prediction was high, the average accuracy of prediction was within the range of 80% to 90%. Increasing the number of predictors did not substantially improve AUC and prediction accuracy. Again, regarding the trade-off between model performance (here, accuracy and AUC) and model size (ie, the number of predictors used), models with 4 to 5 predictors were optimal in terms of the balance between good accuracy and model complexity. Analytical thinking score, positive emotions, discrepancy, causation, drives, first-person singular pronouns, and cognitive processes were the most frequently occurring predictors among the best-performing discriminant models.

Further analysis was conducted on a subset of participants (43/105, 40.9%) who responded to the single-item mental health self-rating immediately after the chat session (49/165, 29.7%). On the basis of language use patterns, a binary classification procedure was able to distinguish those who rated their current mental health status as *poor* from those who rated their health as *fair* to *excellent* (2-5 out of 5) with high discrimination accuracy (AUC 0.95, general accuracy 85.7%, and average accuracy 88.7%), showing a better prediction of participant’s mental health status shortly after a chat session.

All participants who had engaged in the text-based counseling service since August 2019 were contacted in July 2020 and asked to respond to the single-item rating of their mental health. This increased the number of responses from 49 to 165. Binary classification of this expanded data set yielded an acceptable accuracy rate (AUC 0.73); however, this was not as high as that for the participants who rated their mental health immediately after counseling had occurred. We interpret this as an encouraging indication that language use patterns are robust predictors of mental health status and that a larger data set of mental health ratings recorded immediately after counseling would likely yield excellent classification based on language use patterns. All analyses were rerun with the custom Australianized dictionary, but this did not improve the accuracy for any of the models.

**Table 13:** Best 5 discriminant models for discriminating poor response to self-rated mental health from other ratings (fair to excellent).

Number of predictors and predictor names		AUC^a^	General accuracy (%)	Average accuracy (%)	F1 score
**1**					
	Analytical thinking score	0.59	79.4	84.3	0.56
	Discrepancy	0.59	79.4	82.7	0.56
	Insight	0.53	77.6	53.5	0.49
	Present focus	0.51	79.4	75.9	0.55
	Causation	0.50	79.4	82	0.55
**2**					
	Analytical thinking score+other words	0.66	79.4	88.4	0.55
	Discrepancy+drives	0.64	79.4	87.2	0.55
	Clout score+social processes	0.63	79.4	87.2	0.55
	Analytical thinking score+emotional tone score	0.62	79.4	85.9	0.56
	Analytical thinking score+positive emotions	0.62	79.4	85.8	0.56
**3**					
	Positive emotions+discrepancy+personal concerns	0.70	81.2	89	0.63
	Analytical thinking score+other words+drives	0.69	79.4	86.5	0.55
	Analytical thinking score+clout score+other words	0.68	76.4	88.8	0.45
	Analytical thinking score+positive emotions+other words	0.67	79.4	86.1	0.56
	Analytical thinking score+positive emotions+discrepancy	0.66	80	88.6	0.59
**4**					
	Analytical thinking score+other words+cognitive processes+drives	0.72	78.2	87.5	0.52
	Analytical thinking score+positive emotions+discrepancy+personal concerns	0.71	78.2	89.2	0.52
	Positive emotions+causation+discrepancy+personal concerns	0.70	77.6	90	0.49
	Analytical thinking score+clout score+first-person pronouns+positive emotions	0.70	80	88.8	0.59
	Analytical thinking score+positive emotions+discrepancy+drives	0.70	78.2	87.6	0.52
**5**					
	Analytical thinking score+positive emotions+causation+discrepancy+drives	0.73	80.6	87.9	0.60
	Analytical thinking score+clout score+positive emotions+social processes+other words	0.72	80	88	0.59
	Analytical thinking score+emotional tone score+discrepancy+other words+personal concerns	0.72	78.8	87.9	0.54
	Analytical thinking score+positive emotions+discrepancy+other words+drives	0.72	80	87.5	0.59
	Clout score+positive emotions+discrepancy+social processes+personal concerns	0.72	81.2	89.7	0.63
21^b^	All predictors	0.45	34.7	32.1	0.30

^a^AUC: Area under the Receiver Operating Characteristic curve.

^b^The best models with 6-20 predictors (total of 75 items) have lower AUC, general accuracy, average accuracy, and F1 score, thus are omitted.

## Discussion

### Principal Findings

This study aimed to determine whether language use patterns during the course of text-based counseling with a human therapist could be used to predict mental health status. Computational linguistic techniques were used to explore predictive relationships between language use patterns and the participants’ underlying psychological presenting problem, which was recorded before the commencement of counseling, and the self-ratings of their current mental health status, which were recorded after counseling had been completed.

The two aims of the study were to investigate whether language use patterns could be used to (1) identify mental health presenting problems in the clients of *Virtual Psychologist* and (2) predict their self-reported mental health status. Our computational analysis was able to predict the top 3 presenting problems (anxiety, depression, and stress) with an accuracy of 80% (Table 11). The analysis was able to discriminate between those top 3 presenting problems with an accuracy ranging from 50% to 70% (Table 12), which was above the chance level. For the prediction of mental health status as determined by responses to the question, “How would you rate your mental health now?*”* the average accuracy of prediction was good, ranging from 80% to 90% (Table 13).

### Language Use Patterns Can Be Used to Accurately Classify Presenting Problem and Future Mental Health Status

The findings suggest that language use patterns are useful indicators of mental health presenting problems and also predictive of future mental health status. We were able to use linguistic patterns to discriminate the top 3 presenting problems from the remaining pool of 17 presenting problems. This binary classification was able to separate participants with high accuracy. This finding is consistent with previous studies that have reported that depression has linguistic markers, such as increased use of first-person personal pronouns [40-44] and negative emotion words [51]. This study extends past findings by examining language use in a sample of participants with clinically significant presentation, who were receiving text-based counseling. In addition, the approach used here confirms the viability of using text-based counseling chat logs to enable computational linguistic analyses to determine the type of presenting problem.

The predictive analysis was successful in classifying the participants based on their self-ratings of mental health. Binary classification yielded high accuracy in identifying those participants who rated their mental health as *poor* following counseling versus those who rated it as *fair* to *excellent*. This finding provides compelling evidence that linguistic patterns are accurate and robust predictors of future mental health status. Our results support existing evidence that there are linguistic markers related to reductions in symptom severity and improved treatment outcomes [51-53]. To further improve accuracy, we recommend measuring participants’ mental health in a standardized way to reduce variability introduced, for example, by differences in how long after the completion of counseling, the mental health status was measured; however, we acknowledge the difficulties in implementing these recommendations in a real-world clinical context.

As shown in Tables 11 and 13, several linguistic parameters emerged in both the best-performing models for predicting the presenting problems and self-rated mental well-being: first-person singular pronouns, emotional tones, and drives. These linguistic indicators seem to be more related to certain mental problems than other indicators found in existing studies conducted using other approaches [40-44,51-53]. Therefore, our results are consistent with those of previous studies. However, similar to many other computational approaches, one of the disadvantages of using discriminant analysis to find the best-performing prediction model with specific parameters is that, sometimes, it is difficult to interpret why certain parameters emerge as important predictors.

Unlike the study by Seabrook et al [55], the use of a customized dictionary did not improve the accuracy of our analyses. It is unclear why we did not observe similar improvements in depression identification as reported by those authors. There are several important differences between the 2 studies regarding the participant populations and methods of data collection. Seabrook et al [55] recruited participants from a younger age range, likely from urban areas; analyzed Facebook and Twitter status updates; and related these to the scores from a mood-tracking app that their participants downloaded and used. This differs markedly from the farmers recruited in this study, who were engaged in text-based psychological counseling. It is possible that the counseling context is less likely to elicit Australianism, such as slang and other colloquialisms, than posts on social media.

### Differentiating Anxiety From Depression From Stress Is Difficult

Our observation that the models successfully differentiated the top 3 presenting problems from the rest, but were less accurate in differentiating among the top 3 presenting problems, requires explanation. This suggests that the top 3 presenting problems (anxiety, depression, and stress) share common features. This commonality may refer to both the linguistic patterns that individuals with these conditions use and the psychological symptoms that they exhibit. For instance, all 3 problems are likely to affect mood and motivation. Depression and anxiety are known to be highly comorbid conditions. Indeed, 45.7% of individuals with lifetime major depressive disorder also had a lifetime history of ≥1 anxiety disorders [67]. Depression and anxiety also commonly coexist [68]. Furthermore, stress is a response to pressures or threats, whereas anxiety may manifest as a reaction to the stress. Anxiety may not have a clear cause, and as a result, can last longer and be more difficult to treat, but at the time of presentation, both may be affecting the individual. Recall that our participants were recruited during the combined unprecedented events of the Australian bushfire season of 2019 to 2020 and the global COVID-19 pandemic. It seems entirely valid that it may not be possible to statistically differentiate anxiety, depression, and stress from one another because a sizable proportion of participants may have copresented with 2 or all 3 of these mental health problems simultaneously. Another explanation is that it is unknown whether these conditions can be differentiated using default LIWC categories. This is the first study to attempt to differentiate depression from anxiety from stress using linguistic patterns as computed by LIWC. Improved differentiation might be possible by refining the analysis to count the words with the strongest predictive power to separate one condition from the other (ie, going to a finer level of resolution than the coarse LIWC category), as has been demonstrated elsewhere [54]. These possibilities await to be tested in future studies.

### Implications for Practice

The potential applications of an accurate, scalable approach to mental health are far-reaching, with implications for early screening and targeted interventions. Mental disorders are a leading cause of disability worldwide, with enormous economic consequences including lost productivity, employee absenteeism [69], and additional strain placed on carers [70] and health systems [71]. The economic costs owing to lost productivity and absenteeism, even in the case of mild depression, are estimated to be AUD $8 billion (US $5.68 billion) per annum [69]. Although natural language processing of electronic health records is increasingly being used to study mental illness [72], case notes written by therapists and clinicians do not capture the implicit nuances present in the language use patterns of their clients. Thus, they do not lend themselves to the types of predictive analyses described here. Being able to predict future mental health status would enable proactive and early identification of at-risk individuals and bolster harm minimization efforts. On the basis of the linguistic analyses and predictions introduced here, an automated application could be developed to run in the background after each text-based counseling session and output the possible presenting problems and mental well-being status. This could be useful for clinical psychologists to screen at-risk individuals at an early stage and provide subsequent intervention if needed. Thus, such an application would have the potential use of an assistive tool for clinical psychologists. The ultimate goal of such studies is to accurately predict which individuals are at risk of mental health problems (including suicide) so that mental health professionals can intervene and save that person’s life. The present data offer the tantalizing possibility that text-based predictors of mental health status may enable large-scale automatic screening of mental illness and identification of at-risk individuals in the not-too-distant future.

### Limitations and Future Directions

This study has several limitations. First, language use patterns were related to the participants’ presenting problems and self-rated mental health status, but no neuropsychological assessments were administered. Given that our ultimate aim was to identify individuals at risk of clinically significant presentation, it would be advantageous to be able to relate language use patterns to standardized measures of psychological function.

Second, although three-fourth of the participants completed multiple sessions of counseling, the data set only permitted us to relate their language use patterns to presenting problems (recorded before the commencement of treatment) or self-reported mental health status (recorded after they had received counseling). Given that changes in language use have been observed during the course of treatment and these changes have been linked to treatment outcomes [73-75], it would be useful to also track such changes longitudinally when trying to determine a client’s presenting problem. In addition, changes in language use could also be predictive of responsiveness to treatment and future mental health status. For example, increasing use of reflexive language and decreasing use of external language in therapeutic conversation have been associated with better therapeutic outcomes [73].

Third, although the size of the analyzed sample was considerable, the response rate for the self-rating of mental well-being was relatively low (165/773, 21.3% of the sessions). Future replications using similar approaches would benefit from larger data sets, which will increase statistical power and support the detection of significant associations between increased number of variables.

To address these limitations, future studies should include standardized neuropsychological assessments and pharmacological management histories. Ideally, these should be administered at multiple time points during the course of the study to measure changes in symptom severity and how they are reflected in the changes in language use.

### Conclusions

This study suggests that language use patterns during the course of text-based counseling are robust predictors of mental health status in farmers living in rural and remote communities. Linguistic patterns can be used to accurately assign individuals into one of the top 3 presenting problem categories. They can also differentiate those top 3 presenting problems from the pool of other presenting problems via binary classification. If replicated in other samples, computational linguistic analyses may be applied to big data approaches for mental health screening at the population level, providing insight into the linguistic patterns underlying the mental health needs of Australians and improving the speed and scale of identification of at-risk individuals. We were also able to accurately predict future mental health status (as measured by self-ratings) based on linguistic patterns. This technique can potentially provide a sensitive measure of future mental health status that may be used as an early indicator of being predisposed to mental health conditions such as depression, anxiety, and stress.

Text-based counseling serves an important treatment function and has the potential to span great distances to provide e-mental health services to areas where service capacity is lacking. Although text-based communication has limitations (slower than vocal exchanges, faceless, and impersonal), for some segments of the population, it is appealing because of those limits rather than in spite of them (low bandwidth and perceived as offering space and privacy). This study contributes to the understanding of the best approaches for using technology to promote mental well-being and identify individuals at risk of mental health problems.

## Appendix

Multimedia Appendix 1 Australianized Linguistic Inquiry and Word Count dictionary.

## Abbreviations

AUC: Area under the Receiver Operating Characteristic curve
LIWC: Linguistic Inquiry and Word Count
