# Supplementary material for: Predicting Mental Health Status in Remote and Rural Farming Communities: Computational Analysis of Text-Based Counseling
Source: JMIR Form Res. 2022 Jun 21;6(6):e33036. doi: 10.2196/33036 (PMC9257613; doi:10.2196/33036)
Supplement: Multimedia Appendix 1 [file formative_v6i6e33036_app1.docx]

Australian spellings added to LIWC.

| **LIWC Category** | **Added** | **Equivalent in LIWC** |
| --- | --- | --- |
| Verbs (Category 20) | travelled | traveled |
|  | travelling | traveling |
| Adjectives (Category 21) | crueller | crueler |
|  | cruellest | cruelest |
|  | defenceless | defenseless |
|  | foreseeable | forseeable |
|  | grey | gray |
|  | mouldy | moldy |
|  | unsavoury | unsavory |
| Comparatives (Category 22) | crueller | crueler |
|  | cruellest | cruelest |
| Affect (Category 30) | agonise | agonize |
|  | crueller | crueler |
|  | cruellest | cruelest |
| Posemo (Category 31) | splendour | splendor |
| Negemo (Category 32) | agonis* | agoniz* |
|  | crueller | crueler |
|  | cruellest | cruelest |
|  | dishonour | dishonor |
|  | unsavoury | unsavory |
| Anger (Category 34) | crueller | crueler |
|  | cruellest | cruelest |
| Sad (Category 35) | agonis* | agoniz* |
| CogProc (Category 50) | sceptic* | skeptic* |
| Insight (Category 51) | sceptic* | skeptic* |
| Tentat (Category 54) | sceptic* | skeptic* |
| Bio (Category 70) | gonorrhoea | gonorrhea |
|  | gynaecolog* | gynecolog* |
| Health (Category 72) | gonorrhoea | gonorrhea |
|  | gynaecolog* | gynecolog* |
|  | orthopaed* | orthoped* |
|  | paediatr* | pediatr* |
| Sexual (Category 73) | gonorrhoea | gonorrhea |
| Drives (Category 80) | actualis* | actualiz* |
|  | defence* | defense* |
|  | dishonour* | dishonor* |
|  | neighbour* | neighbor* |
| Affiliation (Category 81) | neighbour* | neighbor* |
| Achieve (Category 82) | actualis* | actualiz* |
|  | fulfil* | fulfill* |
| Power (Category 83) | defenceless | defenceless |
|  | dishonour | dishonor |
| Reward (Category 84) | fulfil* | fulfill* |
| Risk (Category 85) | defence | defense |
| FocusPast (Category 90) | travelled | traveled |
| FocusFuture (Category 92) | foreseeable | forseeable |
| Relativ (Category 100) | travelled | traveled |
|  | traveller* | traveler* |
|  | travelling | traveling |
| Motion (Category 101) | travelled | traveled |
|  | traveller* | traveler* |
|  | travelling | traveling |
| Space (Category 102) | kilometre | kilometer |
| Work (Category 110) | finalis* | finaliz* |
| Leisure (Category 111) | travelled | traveled |
|  | traveller* | traveler* |
|  | travelling | traveling |

Australian words added to LIWC.

| **LIWC Category** | **Added** | **Equivalent in LIWC** |
| --- | --- | --- |
| Pronouns (Categories 1, 2, 3, 6) | youse | y'all |
| AuxVerb (Category 12) | gonna | gunna |
| Social (Category 40) | ex-wife |  |
|  | ex-wives |  |
|  | ex-husb* |  |
|  | housemate* | [cf. roommate*] |
|  | youse |  |
| Family (Category 41) | ex-wife |  |
|  | ex-wives |  |
|  | ex-husb* |  |
| Friend (Category 42) | housemate* | [cf. roommate*] |
| Female (Category 43) | ex-girl* |  |
|  | ex-wife |  |
|  | ex-wives |  |
| Male (Category 44) | ex-boy* |  |
|  | ex-husb* |  |
| Percept (Category 60) | mobile | [cf. cellphone] |
| Hear (Category 62) | mobile | [cf. cellphone] |
| Bio (Category 70) | Panadol | [cf. Advil] |
|  | bum | [cf. butt] |
|  | lollies | [cf. candy] |
|  | Paracetamol | [cf. Tylenol] |
| Health (Category 72) | Panadol | [cf. Advil] |
|  | Paracetamol | [cf. Tylenol] |
| Ingest (Category 74) | lollies | [cf. candy] |
| Drives (Category 80) | ATAR | [cf. GPA] |
| Affiliation (Category 81) | housemate* | [cf. roommate*] |
| Achieve (Category 82) | ATAR | [cf. GPA] |
| Power (Category 83) | JP | [cf. judge] |
| Relativ (Category 100) | housemate* | [cf. roommate*] |
| Space (Category 102) | housemate* | [cf. roommate*] |
| Work (Category 110) | ATAR | [cf. GPA] |
|  | Centrelink | [cf. unemployed] |
| Leisure (Category 111) | footy | [cf. football] |
|  | AFL |  |
|  | netball |  |
| Home (Category 112) | housemate* | [cf. roommate*] |
|  | flat |  |
|  | unit |  |
| Money (Category 113) | ATO | [cf. IRS] |
|  | ASD | [cf. USD] |
| Religion (Category 114) | bahai |  |
|  | baha’i |  |
| Informal (Category 120) | abo | [cf. nigger] |
|  | ta | [cf. thks] |
| Swear (Category 121) | abo | [cf. nigger] |
